# On asymmetry of magnetic activity and plasma flow temperature in Jupiter’s magnetosphere

**DOI:** 10.1038/s41598-023-41500-y

**Published:** 2023-09-28

**Authors:** Vitaliy Kaminker

**Affiliations:** Geophysical Institute, University of Alaska Fairbanks, Fairbanks, AK USA

**Keywords:** Space physics, Magnetospheric physics, Plasma physics

## Abstract

Discs of plasma around giant planets are natural laboratories that contain within mechanisms of transferring and keeping energy into the plasma and magnetic field system. Various missions to Jovian planets observed that expansion of plasmadiscs is not adiabatic and plasma temperature is increasing with radial distance. Magnetometer measurements from Juno mission were examined to determine plausibility of turbulent fluctuations as the plasma heating mechanism. Extensive azimuthal map of magnetic activity in Jupiter’s nightside plasmadisc is presented. Observations show that magnetic activity is distributed asymmetrically, with active and quiet regions. This is similar to the asymmetrical distribution of activity observed in Saturn’s magnetosphere. However, comprehensive study of temperature measurements showed that the only systematic change of temperature in magnetospheres of giant planets is in the radial direction. Observed breakfrequency in the magnetometer time series is systematically greater than the ion cyclotron frequency. Examination of the power spectrum points to that the kinetic energy of the corotating plasma as a source of increase of plasma temperature. This study shows that turbulent fluctuations themselves are not good candidates as a plasma heating mechanism. External pressure fluctuation however, can be used to convert kinetic energy of the plasma flow into thermal.

## Introduction

Volcanic activity on Jupiter’s moon Io expels neutral gas at the rate of about $1\ {\textrm{ton/s}}$^1,2^. Part of the neutral population is ionized, forming a plasma torus consisting of oxygen and sulfur ions. Heavy ions are picked up by Jupiter’s strong rotating magnetic field, causing plasma to flow around the planet^3^. Moving plasma expands radially forming the plasmadisc. The tilt of Jupiter’s magnetic dipole causes magnetic field and plasmadisc to wobble. Here measurements and remote sensing of emissions of wobbling plasmadisc are described with respect to the centrifugal equator^4^.

Observations on various missions to the giant planet showed that Jupiter’s plasmadisc expands nonadiabatically. Temperature of plasma increases with increase in the radial distance. Magnetosphere of a giant planet is a natural laboratory that contains within a mechanism of transferring and keeping energy into the plasma fluid and magnetic field system. One suggestion for the mechanism of an increase in plasma temperature in Jovian magnetospheres is heating of plasma fluid with turbulent oscillations.

Turbulent flow consists of eddies on different scales. Turbulent power is introduced to the system at the stirring scale^5^. Energy then cascades down from larger eddies at lower frequencies to smaller eddies with higher frequencies. The cascade is local and one way. Energy transfers from larger scales to neighboring smaller scales without skipping across the range. A major marker on the power spectrum of plasma fluid is the scale of gyration of ions in the magnetic field. At low frequency large fluctuations fluid dynamics is governed by magnetohydrodynamic (MHD) principles where ion fluid motion is associated with fluctuations of the magnetic field a “frozen in” condition. In that subrange power spectrum decays at Kolmogorov rate $P(f) \propto f^{-5/3}$^6^. At frequencies higher than gyrofrequency, in the kinetic (KAW) subrange, variations of ion velocities and magnetic fluctuations decouple. In this subrange spectral index changes to roughly $P(f) \propto f^{-7/3}$, resulting in a steeper decay. Turbulent power cascades from mixing scale, through MHD subrange to gyrofrequency and then continues to cascade through the kinetic subrange.

In this study magnetic activity in Jupiter’s nightside plasmadisc and power spectrum of magnetic fluctuations are examined to determine plausibility of turbulence as a heating source. Methodology of magnetometer time series measurements and systematic power spectrum analysis are presented in Section "Magnetometer measurements". An azimuthal map of magnetic activity in Jupiter’s nightside magnetosphere is presented in Section "Local time distribution of magnetic activity". Examination of power spectrum features and plasma temperature estimate is presented in Section "Examination of the power spectrum of moving plasma and estimation of plasma temperature". Discussion of results is in Section "Discussion".

## Magnetometer measurements

**Figure 1:**
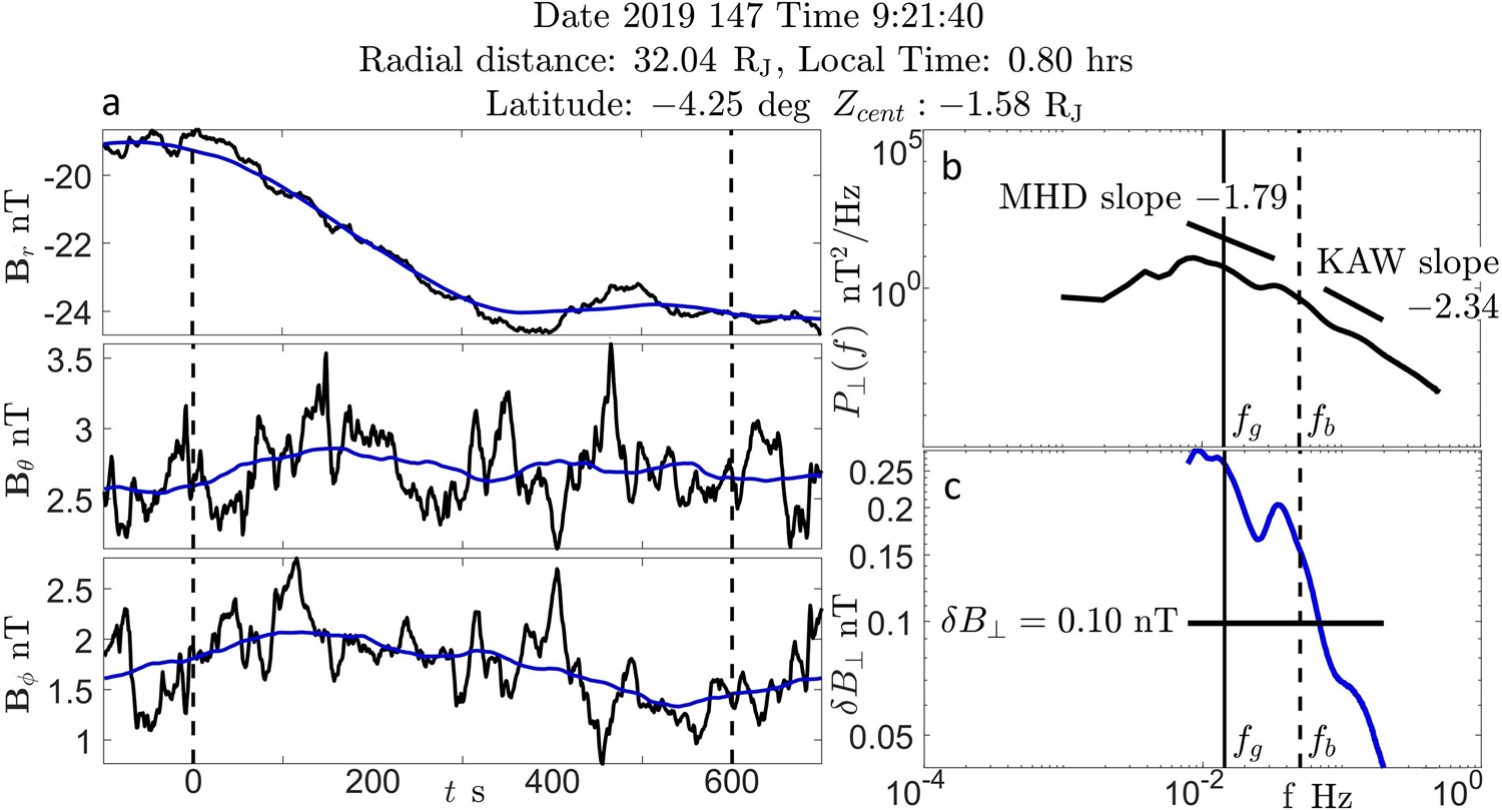
(**a**) Magnetometer time series of the $10\ {\textrm{min}}$ sample window. Blue line is the $200\ {\textrm{s}}$ centered moving average. Area outside dashed lines is a buffer for a moving average sliding window. (**b**) Power spectrum of perpendicular fluctuations. (**c**) Perpendicular magnetic field fluctuation in frequency space.

Time series of the magnetic field in radial Jupiter magnetic (VIP4) coordinates^7^ measured by magnetometer instrument on Juno spacecraft^8^ were analyzed ﻿in $10\ {\textrm{min}}$ window intervals with $1\ {\textrm{s}}$ resolution. This allows to observe plasma with similar properties within the sample and provides a consistent high temporal resolution of magnetometer time series. The main magnetic field $\textbf{B}_0(t)$ was calculated from moving average of the magnetometer time series^9^ (blue line in Fig. 1a). The fluctuation of the magnetic field is then calculated as $\delta \textbf{B}(t) = \textbf{B}(t) - \textbf{B}_0(t)$. Parallel perturbation of the magnetic field is found by $\delta \textbf{B}(t)_\parallel = [\delta \textbf{B}(t)\cdot \hat{\textbf{n}}(t)]\hat{\textbf{n}}(t)$ where $\hat{\textbf{n}}(t)$ is a unit vector in the direction of $\textbf{B}_0(t)$. Perpendicular perturbation was then calculated as $\delta \textbf{B}(t)_\perp = \delta \textbf{B}(t) - \delta \textbf{B}(t)_\parallel$.

Power spectrum is calculated from a time series of $\delta \textbf{B}_\perp$ components with Continuous Wave Transform using Morlet wavelet^10^.

$$\begin{aligned} P(f) = \frac{2}{N\Delta t}\sum ^{N}_{i=1}\Delta t \left| W_i(t_i,f)\right| ^2 \end{aligned}$$

Where $W_i(t_i,f)$ is a Morlet wavelet, $\Delta t$ is the time series step size, *f* is a sampling frequency and *N* is a number of frequency samples. Total power density is then a square root of the sum of squares of the power spectrum of vector components. Perpendicular perturbation of the magnetic field in frequency space is calculated from the power spectrum as $(\delta B_\perp )^2 = P(f)f$^11^ and then averaged across frequency range to get value for the sample window (Fig. 1c). Latitude off the centrifugal equator $lat_{cent}$ was calculated as in^4^ and then distance from the centrifugal equator is calculated as $Z_{cent} = r\sin (lat_{sysIII}-lat_{cent})$. Where $lat_{sysIII}$ is latitude in planet’s rotating frame^3^ and the radial distance is denoted as *r* in Jupiter radii.

**Figure 2:**
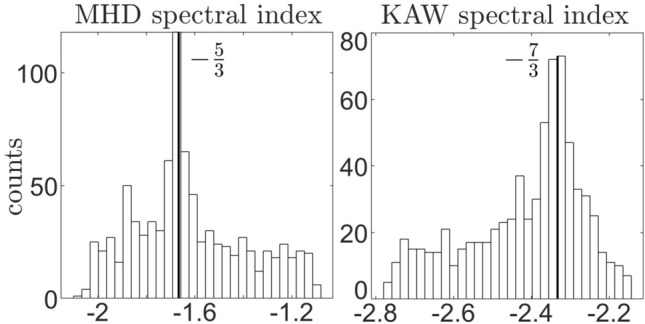
Powerlaw spectral index distribution in MHD and KAW subranges separated by $f_b$ using a slope fitting subroutine. Samples are taken in $r \in [5, 40]\ {\text{R}_\text{J}}$, $Z_{cent} \in \pm 1\ {\text{R}_\text{J}}$ in all sampled local time.

MHD and kinetic subranges are separated by scales of gyration of ions in the magnetic field. In this study gyrofrequency and frequency of the break in the power spectrum were found independently for each $10\ {\textrm{min}}$ sample. Gyrofrequency is calculated as $f_g = \frac{qB}{2\pi m_i}$. Frequency of the break in the power spectrum was determined with the subroutine employing slope spectral index fit to the expected power law decay (Fig. 1b). Power spectrum was examined for a break in the interval $f \in [10^{-2},\ 1.5\times 10^{-1}]\ \ {\textrm{Hz}}$ in $10^{-2}\   {\textrm{Hz}}$ steps. Slopes were fitted to the power spectrum to determine subrange indices and then compared to the expected power law decay rate in the subrange ($-5/3$ for MHD and $-7/3$ for KAW). MHD slope was fitted in the subrange $\left[ 7\times 10^{-3}\  {\textrm{Hz}},\ f_b/1.5\right]$ and KAW slope was fitted in the subrange $\left[ f_b\times 1.5,\ 2\times 10^{-1}\  {\textrm{Hz}}\right]$. Distribution of spectral indices in MHD and KAW subranges separated by the breakfrequency found in the power spectrum samples is shown in Fig. 2. Some spread in slopes shows that simply not all fluctuations in the magnetosphere are due to turbulent cascade.

**Figure 3:**
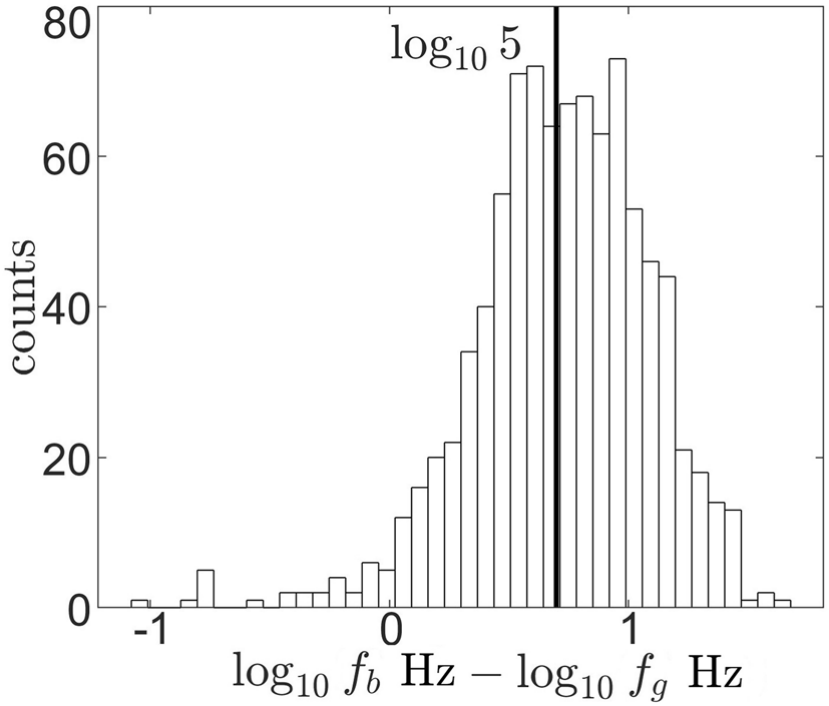
Comparison of gyro and break frequencies in samples from Fig. 2.

Differences between observed frequencies of the break in the power spectrum and ion cyclotron frequencies in $10\ {\textrm{min}}$ samples are shown in Fig. 3. There are a few samples, where found breakfrequency was lower than the gyrofrequency. However, in the vast majority of cases breaks in the power spectrum of moving plasma in the Jupiter magnetosphere are observed at frequencies that are higher than ion cyclotron frequencies. Similar phenomenon was observed in the solar wind^12^, where breakfrequency was found to vary between $f_g<f_b<10f_g$. In cases when breakfrequency was not found by the subroutine it was estimated by the median value $f_b = 5f_g$ (not included in the Fig. 3). The systematic difference between the scales of gyration of ions and scales of observed break in the power spectrum is examined in more detail in Section "Examination of the power spectrum of moving plasma and estimation of plasma temperature".

It is important to note that often in literature^13,14^ (and let’s not forget motivation for this study^15^) gyrofrequency is calculated as $\omega _g = \frac{qB}{ m_i}$. This however is a calculation of angular velocity which has units of $\ {\textrm{rad/s}}$ and should not be plotted on the $\  {\textrm{Hz}}$ axis. Interestingly, unitless $2\pi f_g\sim 6f_g$ in Jupiter’s magnetosphere would put this in the expectation range in Fig. 3. So this then estimates a break in the power spectrum in Jupiter’s magnetosphere in the right ballpark for a wrong reason.

## Local time distribution of magnetic activity

**Figure 4:**
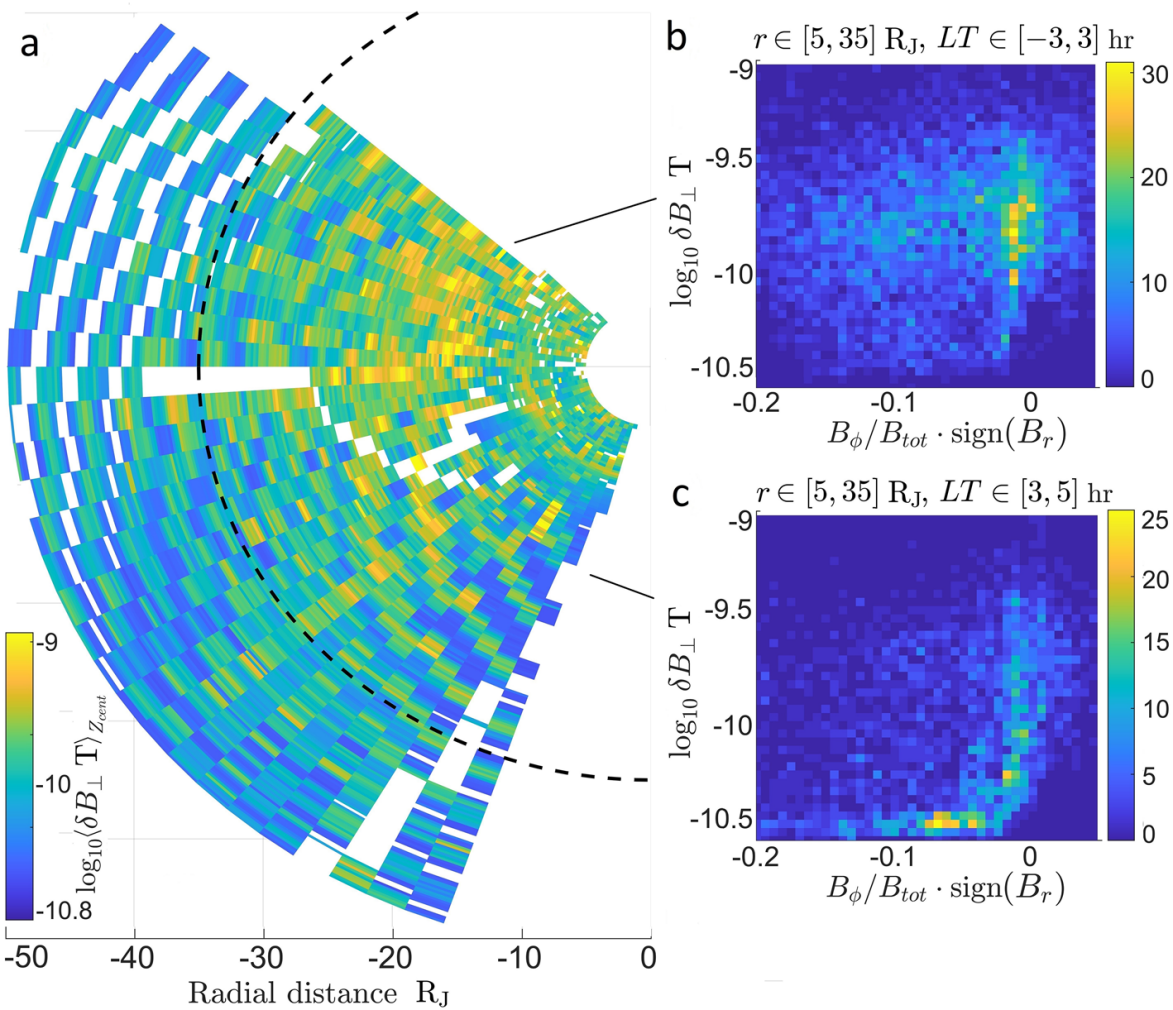
(**a**) Equatorial map of perpendicular magnetic fluctuations. Values of $\delta B_\perp$ are averaged over $Z_{cent} \in [-10, 10]\ {\textrm{R}_{\textrm{J}}}$. Dashed line is $r = 35\ {\textrm{R}_{\textrm{J}}}$. Color bar shows an order of magnitude. (**b**) 2D histogram of magnetic fluctuation vs azimuthal bend of the magnetic field for the region $r \in [5, 35]\ {\textrm{R}_{\textrm{J}}}$, $LT \in [$-$3, 3]\ {\textrm{hrs}}$. (**c**) 2D histogram of magnetic fluctuation vs azimuthal bend of the magnetic field for the region $r \in [5, 35]\ {\textrm{R}_{\textrm{J}}}$, $LT \in [3, 5]\ {\textrm{hrs}}$. Azimuthal bend of the field was calculated as a proportion of the $B_\phi$ component of the magnetic field with respect to the total strength of the field and multiplied by a sign of the $B_r$ component. Colorbar shows number of sample counts.

Equatorial map of magnetic activity of the night side of Jupiter’s magnetosphere is shown in Fig. 4. Map was assembled from perpendicular fluctuation values measured in $10\ {\textrm{min}}$ time series samples (Fig. 1c). Perpendicular fluctuation values were averaged over vertical distance $Z_{cent} \in \pm 10$. Figure 4 demonstrates an asymmetric azimuthal distribution of magnetic activity in the nightside side plasmadisc. Dusk is more active than dawn. Observed part of the plasmadisc was divided into two local time (LT) regions. Amplitudes of magnetic fluctuations were observed with respect to the azimuthal bend of the magnetic field.

Dusk to midnight region $LT \in [-3, 3]\ {\textrm{hrs}}$ is more active with higher amplitude of perpendicular magnetic fluctuations with $\delta B_\perp \sim 10^{-9.75}\ {\textrm{T}}$ (Fig. 4b). Natural topology of the magnetic field inside the plasmadisc is an azimuthal bend back configuration. The observed bend of the magnetic field of the majority of cases in that region is close to neutral. Active magnetic field would tend to oscillate between natural bend back and bend forward configurations. So that the chance observation of the bend of the active magnetic field would probably happen somewhere between the extreme forward and backward deflections. Dawn region $LT \in [3, 5]\ {\textrm{hrs}}$ is much quieter with majority of cases near $\delta B_\perp \sim 10^{-10.5}\ {\textrm{T}}$ with negative bend of the magnetic field (Fig. 4c). The spread of fluctuation magnitude values in the active region is about a half an order of magnitude, while values in the quiet region are tightly packed. This is characteristic of the turbulent environment. Turbulent formations exist on multiple scales where energy cascades from larger scales to smaller ones. So that increase in turbulence not only increases the amplitude of fluctuations but also increases the spread of amplitude ranges. This points to that the activity in dusk to midnight region is indeed turbulent.

This result is similar to what was observed in Saturn’s magnetosphere^15^. Survey of magnetic fluctuations quantified as turbulent heating indicated presence of a quiet $LT \in [3,9]\ {\textrm{hrs}}$ and an active $LT \in [10, 20]\ {\textrm{hrs}}$ regions in Saturn plasmadisc. The dusk-dawn asymmetry of magnetic activity in magnetospheres of Jovian planets must be a result of the interaction of the spinning magnetosphere and the solar wind. This implies that this interaction is a major contributor to turbulence in magnetospheres of Jovian planets. Systematic study of magnetic fluctuations around giant planets^15,16^ surprisingly point to that the magnetic activity is higher in azimuthal regions where the flow in the sheath opposes the natural backward azimuthal bend of the magnetic field rather than the flow in the plasmadisc. It is also surprising to see the effect of interaction of the magnetosphere with solar wind well within $r<35\mathrm \ R_J$.

There is no discernible dependence of magnetic activity on local time beyond $r>35\, {\textrm{R}_{\textrm{J}}}$ within azimuthal regions observed by Juno spacecraft. This study also shows that there is no System III dependence of magnetic fluctuations in the plasmadisc. This differs from results published in^17^. Additional description and figures are in the appendix of this paper.

Extensive examination of moments from the Galileo mission showed that there is no significant local time or System III variation of ion temperature in Jupiter’s plasmadisc and that the only systematic variation is in the radial direction^1^. Similarly no such LT asymmetry of ion temperature in Saturn’s magnetosphere was reported^18^. Which points to that asymmetrically distributed turbulent activity in plasmadisc is not a good candidate for the heating mechanism of plasma in Jovian magnetospheres.

## Examination of the power spectrum of moving plasma and estimation of plasma temperature

### Doppler effect on observed break in the power spectrum

Observation of magnetometer time series showed that break in the power spectrum systematically occurs at frequencies that are higher than the gyrofrequency (Figs. 1 and 3). Fluctuations in a moving medium are described in terms of oscillations as observed in the frame moving with the medium modified by a Doppler shift.

1 $$\begin{aligned} \omega ' = \omega + \textbf{k} \cdot \textbf{v} \end{aligned}$$

In Jupiter’s magnetosphere Doppler shift effect can be written as.

2 $$\begin{aligned} \textbf{k} \cdot \textbf{v} \sim k_\perp v_\perp \end{aligned}$$

Where $\perp$ implies quantity perpendicular to the main magnetic field. Here $v_\perp = v \sin (\theta _{vB})$^13,19^ and $\theta _{vB}$ is the angle between plasma bulk flow velocity and main magnetic field in Jupiter sun orbit (JSO) coordinate system^7^. Here note that measurement of $\delta B$ is a measurement of the amplitude of the oscillation and not wavelength or direction of the wave propagation. This simplified estimate of the dot product assumes that waves detected by the spacecraft are propagating along the direction of the perpendicular component of the bulk velocity, moving towards the spacecraft.

It is reasonable to assume that the wavelength of the perpendicular fluctuation of the field at ion gyrofrequency is comparable to the circumference of ion gyration $k_\perp (f_g)\rho _i \sim 1$ (comparable cycles of motion of ions and magnetic field). In this work it is deduced that Doppler shift is responsible for the difference between observed frequency of the break and gyrofrequency (Fig. 1). Then Eq. (1) can be written in terms of breakfrequency, gyrofrequency and gyroradius.

3 $$\begin{aligned} 2\pi f_b \sim 2\pi f_g + \frac{v_\perp }{\rho _i} \end{aligned}$$

The ion gyroradius is calculated in terms of the ion temperature.

$$\begin{aligned} \rho _i = \frac{\sqrt{m_ik_bT_i}}{eB} \end{aligned}$$

Introducing Doppler shift constant

$$\begin{aligned} c_{ds} = \frac{f_b - f_g}{f_g} \end{aligned}$$

equation (3) can be rewritten as

$$\begin{aligned} 2\pi c_{ds} f_g \sim \frac{v_\perp }{\rho _i} \end{aligned}$$

Substituting for gyrofrequency and gyroradius it can then be solved for the ion temperature

4 $$\begin{aligned} T_i \sim \frac{m_iv_\perp ^2}{c_{ds}^2k_B} \end{aligned}$$

Note that in Eq. (2) a bulk flow in Jupiter’s plasmadisc was assumed to be faster than the wave speed. So that fluctuations of waves moving towards the sensor are observed. In more general situation, the Doppler shift $\textbf{k}\cdot \textbf{v}$ can very well be negative. In that case break in the power spectrum will be observed at frequencies lower than the gyrofrequency $f_b < f_g$. In principle, magnitude of the angle between bulk velocity and wave propagation can be anywhere between 0 and $\pi$. If direction to the source of the wave is known then the more general expression for the ion temperature is

$$\begin{aligned} T_i \sim \left( \frac{\hat{\textbf{n}}_k \cdot \hat{\textbf{n}}_v}{c_{ds}}\right) ^2\frac{m_iv^2}{k_B} \end{aligned}$$

As the angle between $\textbf{k}$ and $\textbf{v}$ approaches $\pi /2$, $f_b$ will approach $f_g$ so that both $c_{ds}$ and $\hat{\textbf{n}}_k \cdot \hat{\textbf{n}}_v$ will become small with canceling effect and as the angle between $\textbf{k}$ and $\textbf{v}$ approaches $\pi$ both $c_{ds}$ and $\hat{\textbf{n}}_k \cdot \hat{\textbf{n}}_v$ will become negative. Also note that $c_{ds} > -1 \forall f_b$ and $f_g>0$.

In absence of a better estimate for $\hat{\textbf{n}}_k \cdot \hat{\textbf{n}}_v$ temperature was calculated using Eq. (4) with cases in the range $f_b> 2f_g$. Here bulk velocity modeled as a corotation velocity up to $20\, {\textrm{R}_{\textrm{J}}}$, After that velocity is $\textbf{v} = \textbf{v}_\phi = 200\hat{\textbf{e}}_\phi \ {\textrm{km/s}}$^18^. Near the planet gyro and breakfrequencies for cases described above increase with increase in the strength of the magnetic field. In samples from magnetometer measurements^8^ where break is beyond the upper bound of the range used by the algorithm $1.5\times 10^{-1}\ {\textrm{Hz}}$ (Fig. 1b) or where break in power spectrum was not found, breakfrequency is estimated by the median value (Fig. 3).

**Figure 5:**
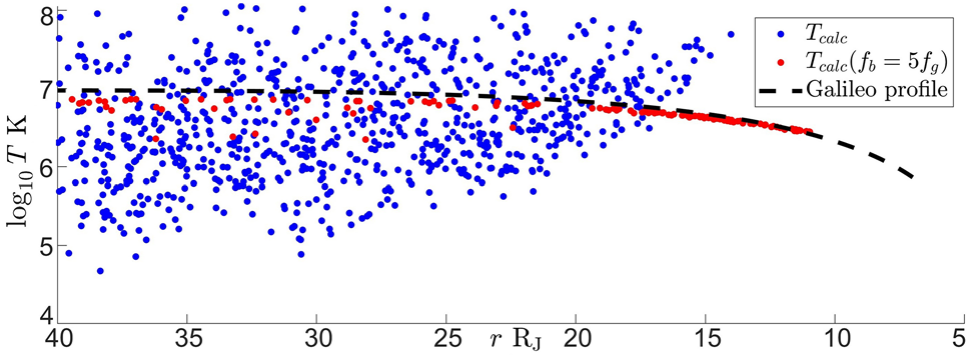
Radial profile of Temperature estimate made with breakfrequency found by the subroutine of ion temperature (blue markers). Temperature estimate made with breakfrequency calculated as $f_b = 5f_g$ (red markers). Samples are taken in $r \in [5, 40]\ {\textrm{R}_{\textrm{J}}}$, $Z_{cent} \in \pm 1\ {\textrm{R}_{\textrm{J}}}$ in all sampled local time. Temperature fit from Galileo mission^18^ (dashed line).

Figure 5 shows distribution of temperature estimates. Temperature calculations made with breakfrequency found by the subroutine are shown with blue markers. These estimates compare fairly well to the plasma temperature fit from Galileo mission^18^ with the spread that is similar to the spread in reported temperature measurements. Temperature calculations made using the median estimate for the breakfrequency are shown with red markers. These estimates land on the Galileo profile. So that if one would not bother to try to find a break in the power spectrum and just calculated it as $5f_g$ then Eq. (4) will give the temperature profile from^18^.

An intriguing possibility is the use of external fluctuations to trigger cascade in moving plasma closer to gyrofrequency. In this case both $c_{ds}$ and $v_b$ in Eq. (4) will decrease. This in principle could allow to convert kinetic energy of the plasma flow into thermal. It is possible that waves generated by magnetic activity in Jupiter’s magnetosphere are used to precipitate a break in the power spectrum closer to gyrofrequency. The amount of kinetic energy transferred to thermal, then depends on the difference between gyro and breakfrequencies. In Jupiter’s magnetosphere this process is inefficient, the spread of the difference between break and gyrofrequencies is about an order of magnitude (Fig. 3). This then results in the natural spread of temperature values^18^ and Fig. 5.

### Doppler effect on general frequency spectra

Assuming that the Doppler effect is similar for fluctuations at different frequencies a more universal relation between observed wave numbers and wave numbers in the bulk flow reference frame can be made. In that case a relation between observed and original frequencies is similar to the relation between break and gyro frequencies.

$$\begin{aligned} \frac{f'-f}{f} \sim \frac{f_b-f_g}{f_g} = c_{ds} \end{aligned}$$

Here the observed frequency of the break in the power spectrum is used as a marker for the Doppler shift of observed wave fluctuations in the rest of the frequency range. So then Eq. (1) can be solved for *k*.

5 $$\begin{aligned} k \sim \left( \frac{c_{ds}}{c_{ds}+1}\right) \frac{2\pi f'}{v (\hat{\textbf{n}}_k \cdot \hat{\textbf{n}}_v)} \end{aligned}$$

Note that Eq. (5) will deviate from Taylor’s hypothesis^20^ when observed frequency is very similar to the original frequency and $c_{ds}\ll 1$, so in the case of a very low velocity or a very long wavelength. It allows for the observation of waves with an arbitrary angle between directions of wave and bulk velocities. So that this enables an observation of incoming wave fluctuations from observed frequency, bulk velocity and the breakfrequency in the power spectrum. Alternatively, if wavenumber features *k*(*f*) in rest frame are known then using observed $f'$ and $f_b$ Eq. (5) can be solved for direction to the source $\hat{\textbf{n}}_k \cdot \hat{\textbf{n}}_v$.

## Discussion

In this work Juno magnetometer measurements were used to examine magnetic activity in Jupiter’s night side magnetosphere and to attempt to determine mechanism responsible for increase of plasma temperature in Jupter’s plasmadisc. Magnetic field observations show an asymmetry of activity between night: dusk and dawn sectors of the magnetosphere. The dusk side is more active than the dawn side. Amplitudes of fluctuations in the active dusk region are about two orders of magnitude greater than in the dawn region. Local time asymmetry of magnetic activity must be a result of interaction of solar wind with magnetosphere of the planet. Surprisingly, observation of magnetic fluctuations show that magnetic activity is higher in azimuthal regions where the flow in the sheath opposes the natural backward azimuthal bend of the magnetic field rather than the flow in the plasmadisc. Equally surprising result is to see the effect of interaction of the solar wind with magnetosphere well within $r<35\ {\textrm{R}_{\textrm{J}}}$.

Similar azimuthally asymmetric distribution of magnetic activity was observed in Saturn’s magnetosphere^15,16^. Survey of fluctuations of the magnetic field from Cassini magnetometer data, quantified as turbulent heating, showed local time dependence of activity in Saturn’s magnetosphere^15^ with active and quiet sites. Azimuthal locations where flow in the sheath is opposite to the flow in the plasmadisc have lower magnetic activity than areas where flow in the sheath and disc are in the same direction. This is opposite to expected Kelvin-Helmholtz instability driven turbulence process. So then Kelvin-Helmholtz vortexes had to be transported to the other side of the giant’s magnetosphere^16^. Examination of magnetic activity in Jovian magnetospheres point to that it is more likely that magnetic fluctuations due to field topology in conjunction with fluid velocity variations play a crucial role in generating turbulent oscillations.

Turbulence was suggested as a possible source of energy needed to heat plasma in Jovian magnetospheres. Estimates of the total power input necessary to counteract an adiabatic cooling of expanding plasmadiscs in Saturn’s and Jupiter’s magnetospheres were done in^18^. However, on closer examination it is unclear what volume was used in calculation of the adiabatic expansion in^18^. There an attempt was made to scale the volume of a shell with change in the magnetic flux at different *L* dipole McIlwain parameters. Volume was calculated as $L^3H$. This volume however does not scale as a disc where plasma fluid is contained. Indeed $R^{3.85}H$ in equation (13) of that publication is not a 3D volume at all, as is required for adiabatic process relation derived from thermodynamic equations for plasma fluid contained in Jupiter’s plasmadisc or coffee contained in a thermos.

If turbulent power is responsible for the increase in plasma temperature and the timescale of transfer of turbulent energy in to ion temperature ”heating” is less than the rotation period of the plasmadisc $\sim 10\ {\textrm{hrs}}$, then one would expect to see a systematic increase in plasma temperature as it passes active regions. However, extensive examination of moments from Galileo showed that there is no significant local time or System III variation of ion temperature in Jupiter’s plasmadisc^1^. Similarly no such local time asymmetry of ion temperature in Saturn’s magnetosphere was reported^18^. Which points to that azimuthally asymmetric turbulent activity in plasmadiscs of Jovian planets is not a good candidate for the mechanism to rationalize a radial increase in plasma temperature.

Magnetohydrodynamic and kinetic subranges are separated by scales of gyration of ions in the magnetic field. It is reasonable to assume that the wavelength of the perpendicular fluctuation of the field at ion gyrofrequency is comparable to the circumference of ion gyration. Examination of power spectrum of magnetometer time series samples shows that break in the power spectrum of moving plasma consistently occurs at frequencies that are on average half an order of magnitude higher than the ion cyclotron frequency. In this work it was deduced that this effect is due to the Doppler shift of observed oscillations. Doppler effect relation can then be used in conjunction with comparison of field wavenumber and inverse of ion gyroradius to express plasma temperature in terms of bulk flow velocity. This then implies that kinetic energy of the plasma flow is the primary source of increase of ion temperature. Turbulent fluctuations themselves are not good candidates for a plasma heating mechanism. External fluctuations however, can be used to convert kinetic energy of the plasma flow into thermal.

Difference between observed frequency of the break in the power spectrum and ion gyrofrequency can be used as a marker for the Doppler shift for the rest of the frequency spectrum. This then enables to modify Taylor’s hypothesis to take into account oscillations of passing wave features. Wavenumber spectrum of the incoming wave in the bulk flow reference frame can be inferred from the observed frequency, bulk velocity and breakfrequency.

## Figures and Tables

**Figure 6:**
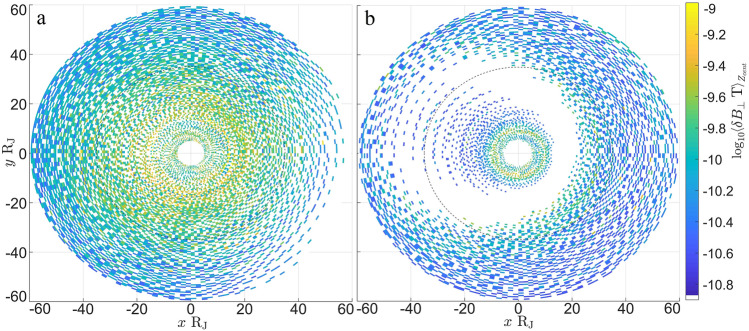
(**a**) Azimuthal map of magnetic activity in System III reference frame averaged over the range $Z_{cent} \in [$-$5, 5]\ {\text{R}_\text{J}}$. (**b**) Azimuthal map of magnetic activity in System III reference frame averaged over the range $Z_{cent} \in [$-10, -$5]\ {\text{R}_\text{J}}$.

**Figure 7:**
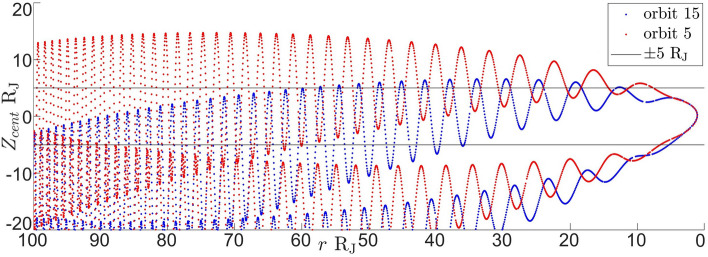
Sample of $Z_{cent}$ for orbits 5 and 15.

## Data Availability

The datasets analysed during the current study are available in the [JUNO MAGNETOMETER JUPITER ARCHIVE] repository^8^, [https://pds-ppi.igpp.ucla.edu/search/view/?id=pds://PPI/JNO-J-3-FGM-CAL-V1.0], doi: 10.17189/1519711].

## Appendix

Azimuthal map of magnetic activity in Jupiter’s rotating reference frame (System III)^7^ is shown in Fig. 6. Figure on the left shows $\delta B_\perp$ values averaged over $Z_{cent} \in [-5, 5]\ {\text{R}_\text{J}}$. There is no discernible evidence of magnetic activity dependence on azimuthal position in the System III coordinate frame within the vicinity of the plasma disc.

Figure on the right shows an example of $\delta B_\perp$ values averaged over $Z_{cent} \in [-10, -5]\ {\text{R}_\text{J}}$. Dark blue values inside the white space are outgoing trajectories moving in negative $Z_{cent}$ direction. So that these $\delta B_\perp$ values are sampled further down in $Z_{cent}$ negative direction, with much lower magnetic activity than in $Z_{cent} \in [-5, 5]\ {\text{R}_\text{J}}$ region. Values surrounding white space are inbound trajectories. The asymmetry of sampling is due to the geometry of Juno’s orbit with respect to the wobbling plasmadisc.

A general state of activity in the plasmadisc can be inferred from the observation of perpendicular magnetic fluctuations outside the disc. Examination of plasma flow attributes however must be done with consideration to variation of plasma properties within the sample. Continuous $10 \mathrm \ hr$ time intervals, $\sim 1$ “wobble” (Fig. 7) will include plasma sample from inside and outside of the plasmadisc with very different properties. For example, the same time series will include locations with and without break in the power spectrum and kinetic subrange present. These time series *will also include* asymmetric sampling of locations beyond $|Z_{cent}| = 5\ {\text{R}_\text{J}}$^17^. This asymmetry will then show up in the power spectrum. There are of course many other, less intricate ways to look at the geometry of the satellite orbit than inspection of the power spectrum of magnetometer time series.
